# Treatment-Resistant Depression in America Latina study: one-year follow-up of treatment resistant depression patients under standard of care reveals insights on quality of life, disability, work impairment, and depressive symptoms

**DOI:** 10.3389/fpsyt.2023.1221746

**Published:** 2023-10-27

**Authors:** Kelen Recco, Gerardo Garcia Bonetto, Christian Lupo, Antonio E. Nardi, Arnulfo Morales, Claudia Becerra-Palars, Sergio Perocco, Alanna Pfau

**Affiliations:** ^1^Instituto de Neurociências Dr João Quevedo, Criciúma, Santa Catarina, Brazil; ^2^Hospital Neuropsiquiatrico Provincial de Córdoba, Córdoba, Argentina; ^3^Centro de Investigación y Asistencia en Psiquiatria, Rosario, Argentina; ^4^Outpatient Clinic for Resistant Depression, Institute of Psychiatry, Federal University of Rio de Janeiro, Rio de Janeiro, Brazil; ^5^Instituto de Seguridad Social del Estado de México y Municipios, Toluca, Mexico; ^6^Instituto Nacional de Psiquiatría “Ramón de la Fuente Muñiz”, Mexico City, Mexico; ^7^Janssen Cilag Farmacêutica Ltda, São Paulo, Brazil; ^8^Janssen, Pharmaceutical Companies, Titusville, NJ, United States

**Keywords:** treatment-resistant depressive disorder, longitudinal study, quality of life, standard of care, patient reported outcome measures, Latin America

## Abstract

**Introduction:**

Depressive Disorders are on the rise worldwide. This is also the case in Latin America (LatAm). Treatment-Resistant Depressive Disorder (TRD) poses additional burden to patients with depression. Impacts quality of life (QoL) and other dimensions, and standard of care (SOC) is insufficient to achieve the desired clinical outcomes. Evidence from LatAm is, however, lacking. The present study was devised as a 1-year follow-up of the SOC in TRD patients in LatAm to explore the burden of TRD.

**Methods:**

This was an observational, multinational, longitudinal study. Patients with clinical diagnosis of TRD in LatAm were included in a 1-year follow-up with SOC. Beyond the Sociodemographic characterization, outcome measures were QoL (EQ-5D-5L), disability (Sheehan Disability Scale - SDS), work productivity (Work Productivity and Activity Incapacity Questionnaire: depression - WPAI:D) and depression severity (Patient Health Questionnaire-PHQ9). Patients were assessed every 3-months and comparison was performed based on change from baseline to each visit and end of study (EOS - 12 months).

**Results:**

Patients averaged 48 (± 13.12) years, mostly female (80.9%) and married/consensual union (42.5%) or single patients (34.4%). Despite the SOC treatment, three-quarters of the patients remained symptomatic at EOS, regardless of the significant longitudinal decrease (*p* ≤ 0.001). Similar trends were found for disability (*p* ≤ 0.001) -82.2% of the patients reporting work/school disruption at EOS-, percentage of work (34%) and activity impairment (40%) at EOS (*p* ≤ 0.001) and only 29.2% of patients with depressive severity “none” at EOS (*p* ≤ 0.001). The results portray the need to improve clinical outcomes in this complex and burdensome disease in LatAm.

**Discussion:**

Here we show that the burden of TRD remains significant in essential dimensions of everyday life at EOS underlining the need for better therapeutic solutions. The improvements in most patients do not provide the desired outcome of return to the state before the condition. Further research should focus on identifying which treatments provide better outcomes in a real-world context.

## 1. Introduction

The impact of Major Depressive Disorder (MDD), a severe form of depression, is well-known and documented in literature. This makes MDD one of the most significant contributors to overall disability (1–3), and one of the priorities to be addressed in the next decade. A major concern regarding MDD is the lack of clinical response in a significant proportion of the patients-more than one third- (4–6) over long periods of treatment with Standard of Care (SOC) (7), resulting in Treatment-Resistant Depression (TRD). TRD is often defined as a failure to respond to two or more antidepressants at adequate therapeutic doses, over an appropriate period of time, within the current depressive episode (8). However, there is not a clear consensus on the definition, and recent efforts have been made to make it more specific and operational (9). This lack of consensus is an obstacle for robust comparison between trials and hinders essential conclusions on the effectiveness of specific therapeutics on this condition (10). A recent consensus guideline has made some strides in addressing this issue by providing practical and insightful recommendations on how to design and implement clinical trials for TRD set on addressing current and future unmet needs, as well as knowledge gaps, so more structured trials should be expected in the near future (11).

SOC usually includes selective serotonin reuptake inhibitors (SSRIs), dual serotonin and norepinephrine reuptake inhibitors (SNRIs) or even antipsychotics and other non-pharmacological therapies such as psychotherapy (9). The efficacy of current treatments in SOC raises concerns since it is far from the desired outcome. Only around 44% of the TRD patients achieve clinically meaningful response (12). Improvement may potentially be achieved through the inclusion of novel therapeutics that have been shown to present promising results (13–15). Although the diversity of therapeutics and treatment combinations is increasing, the proportion of patients not achieving response or response without remission can be higher than previously mentioned, even in countries such as Italy (16). Moreover, there is no consensus on the best therapeutics to improve clinical outcomes, and real-world studies have provided extensive evidence on this topic (17). Augmentation and combination are frequently used. In some cases, the added potential of including additional therapeutics in the treatment regimen have even proven to be counterproductive-such is the case of atypical antipsychotics-compared to switching to another antidepressant (18). Also, due to the uncertainty of the condition and the possibility of life-threatening outcomes, even in most cases in which response is not obtained, treatment changes seem to be less frequent than maintenance (17). Recent research on new treatment strategies shows the potential and promise of new treatments, such as repetitive transcranial magnetic stimulation (19), the magnetic seizure therapy as an alternative to electroconvulsive therapy (20), monoaminergic reuptake inhibitor – toludesvenlafaxine- (21), glutamatergic modulators (22–25), deep brain stimulation (26), and others currently being explored to add to the therapeutic arsenal for TRD treatment (27). The current advances in cognitive neuroscience have also brought forward new approaches to managing depression and anxiety, which should contribute to more focused and individualized therapies (28).

As in other conditions, namely psychiatric disorders, clinical outcomes are no longer the only focus of research and intervention. In a context in which patient-centered care is becoming increasingly more present, models that include shared decision-making can improve engagement and subsequent outcomes even in complex populations such as war veterans (29). These models address an identified unmet need concerning patient education (30). TRD patients have more severe clinical presentation than other MDD patients, are more prone to suicidality, have a higher proportion of comorbidities, have higher healthcare resource utilization, resulting in higher economic burden of the disease, and negative outcomes on quality of life (4, 31, 32). The added impact on healthcare resource utilization in patients with TRD, matched with non-TRD patients, have been demonstrated in a population-wide study (33). TRD impacts several dimensions of daily living, such as work impairment, disability and other Patient Reported Outcomes (PROs) (4, 34–36). In this regard, the impact on instrumental activities of daily living (IADLs) should be considered, which should desirably be returned to the state previous to the episode of MDD/TRD. Previous real-word evidence has shown that outcomes of MDD/TRD are significant in dimensions such as quality of life and disability, and this is more evident in non-responders to treatment (16). The significanlty high indirect effect of absenteeism should not be discarded, as it accounts for 70% of the costs associated with a major depressive episode in a study in Belgium (37).

TRAL (Treatment-Resistant Depression in America Latina) was a multinational study aiming primarily to estimate the prevalence of TRD in LatAm. TRAL generated much needed real-world epidemiological data on MDD and TRD patients under SOC treatment at regional reference sites on multiple dimensions-from clinical to economic, humanistic, quality of life and work productivity dimensions (4, 34, 36, 38). A longitudinal 1-year follow-up of TRD patients under SOC at reference centers in LatAm (phase 2) followed the epidemiological design. Some previously published results from phase 2 of the TRAL study (12) focused more on a clinical characterization and response to SOC of TRD patients, highlighting the unmet needs in TRD treatment in LatAm-mostly associated with low treatment response rates and the proportion of relapses with current SOC. Hence, this is not exclusive toLatAm, as recent efforts in other regions-such as Europe-provided evidence to address the same unmet need (17).

This paper reports on the results obtained from the PROs (quality of life, disability and work impairment) of TRD patients over a 1-year follow-up, as well as some clinical characterization of depression severity. The main objective was to assess the burden of the disease using the Instrumental Activities of Daily Living (IADLs) and work-related activities-beyond the clinical characterization of TRD. By addressing the objectives, TRAL has the potential to close the knowledge gap in LatAm concerning outcomes of SOC. Also, it aims to inform all stakeholders, ensuring that strategies for the regions can be set on solving some of the unmet needs affecting TRD patients in LatAm.

## 2. Materials and methods

### 2.1. Study design and population

TRAL was a multicenter, multinational, observational study conducted in a real-world setting (October 2017–December 2018) which included regional psychiatric sites from Argentina, Brazil, Colombia and Mexico. A thorough description of TRAL’s methodology can be found in previous publications of the project (12, 39). This study followed a similar approach to other real-world studies set on characterizing the clinical and non-clinical outcomes of TRD based on the routine standard of care (16–18, 33, 40). The importance of real-world evidence in this context should not be understated given the differences between clinical trials and the routine management of TRD patients, in which real-world evidence better depicts the treatment unmet needs. The present analysis refers to the longitudinal analysis of PROs and a depression severity scale over a 1-year follow-up of TRD patients under SOC. SOC was considered as a diagnostic and treatment process that a clinician followed for a certain type of patient, illness, or clinical circumstance according to best available evidence, and that is used in the routine clinical practice. Regional centers were all reference psychiatric treatment sites, as seen in previous publications. From 430 TRD patients clinically diagnosed, 420 patients were included in the follow-up. A full list of inclusion and exclusion criteria for the study has been previously published (12, 39). For the purpose of this study, TRD was defined as failure to respond to two or more antidepressants at adequate therapeutic doses, over an appropriate period of time, as assessed by in the routine clinical setting. Inclusion criteria included a diagnosis of TRD based on adequate follow up and treatment with at least 2 antidepressants, and without complete response to treatment [based on Montgomery – Åsberg Depression Rating Scale (MADRS) (37)]. Diagnosis of TRD was based on DSM-V criteria, and on MINI, and considered the study’s TRD definition.

### 2.2. Data and assessments

Depression severity was assessed with the Montgomery – Åsberg Depression Rating Scale (MADRS) (41), a 10-item scale with good discrimination between responders and non-responders to antidepressants, to determine response to SOC over a 1-year time span. Based on MADRS, the following variables were calculated: (a) Change of MADRS score from visit 1 (%) – The following formula must be considered:

$\begin{array}{l} \mathrm{MADRS}\;\mathrm{change}=\left(\begin{array}{l} \mathrm{MADRS}\;\mathrm{score}\;\mathrm{at}\;\mathrm{visit}\;i\\ -\mathrm{MADRS}\;\mathrm{score}\;\mathrm{at}\;\mathrm{visit}\;1 \end{array}\right)\\ \;/\left(\mathrm{MADRS}\;\mathrm{score}\;\mathrm{at}\;\mathrm{visit}\;1\right)*100 \end{array}$

The Patient Health Questionnaire (PHQ-9) was also included to assess depression severity (42, 43). This is a 10-item questionnaire that characterizes the severity of symptoms on a 4-point scale (0 – Not at all, 1 – Several days, 2 – More than half the days, 4 – Nearly every day) relative to a pre-defined time frame, usually the last 2 weeks. PHQ-9 can be scored as ‘None’ (score between 0 and 4), ‘Mild’ (score between 5 and 9), ‘Moderate’ (score between 10 and 14), ‘Moderately severe’ (score between 15 and 19) and ‘Severe’ (score between 20 and 27).

The Work Productivity and Activity Impairment Questionnaire (WPAI:D) was included to assess the impact of the condition in work related activities (44). Questions relate to the last 7 days. Results can be organized in the following dimensions: Percent work time missed due to depression, Percent impairment while working due to depression, Percent overall work impairment due to depression and Percent activity impairment due to depression. For more information, please refer to previous publications (12).

Sheehan Disability Scale (SDS) is a 3-item questionnaire, corresponding to 3 scales, aimed at assessing the level of functional disability from a specific condition (45). Each scale of the SDS questionnaire was recoded according to the following cut-offs: ‘Not at all’ – score equal to 0, ‘Mildly’ – score between 1 and 3, ‘Moderately’ – score between 4 and 6, ‘Markedly’ – score between 7 and 9 and ‘Extremely’ – score equal to 10. Total SDS score was obtained by summing the 3 scales/items that range between 0 (unimpaired) and 30 (highly impaired). A patient that scores 5 or more in any of the scales should be closely monitored since it implies significant functional impairment.

Quality of life was assessed with the EQ-5D-5L questionnaire (46). This is a 5-dimension questionnaire that performs a self-report on Mobility, Self-Care, Usual Activities, Pain/Discomfort, Anxiety/Depression, and a global assessment visual analog 100-point scale. Score were also converted to the EQ-5D-3L score using responses in the EQ-5D-5L index values based on US values set (47).

Sociodemographic and clinical features at baseline were collected and assessed by a physician, while clinical features were again collected every 3 months until the end of the study (12 months from baseline).

Written informed consent was obtained from all participants. The study was approved by local Independent Ethics Committee/Institutional Review Board.

### 2.3. Statistical analysis

Sample size calculations can be found in previous publications (12). 334 TRD patients were necessary, but 387 were recruited assuming a 15% dropout.

The overall TRD sample in LatAm included 420 patients. From 1,544 MDD patients, 387 were initially planned to be included, but was later increased to 420. When the required number was achieved, the remaining TRD patients were not included.

To reach the sample size and considering that the sites are specialized in the treatment of mental disorders, an estimated prevalence of 21.7% was assumed. Baseline assessment was performed on a sample of 1,544 MDD patients, intended for an even distribution in each country. However, due to recruitment constraints and lengthy regulatory decisions in Brazil, sample size was adjusted and even distribution was not achieved.

Quantitative variables were summarized as mean, median, standard deviation minimum and maximum, and qualitative variables were summarized as absolute frequency and percentage, overall and by TRD and non-TRD subgroups. Longitudinal comparisons on clinical outcomes were performed with a Generalized estimating equation for a 95% Confidence interval. Correlation was performed with the Spearman’s correlation coefficient in accordance with recommended statistical procedures based on sample size and normality assumptions.

There was no imputation of missing data. Statistical significance was set at 5%. Statistical analysis was performed using SAS^®^ (version 9.4, SAS Institute Inc., Cary).

## 3. Results

### 3.1. Patient disposition and sociodemographic characteristics

From an initial sample of 1,475 MDD patients enrolled in the study in 4 LatAm countries, 430 were diagnosed with TRD based on the criteria defined by protocol, but only 420 patients with TRD were included in the second phase (1-year follow-up) of the study [for the reason for non-inclusion, please consult previous TRAL publications (12, 39)]. Of these, around 75% completed the 1-year follow-up due to previously disclosed factors.

Sociodemographic characterization suggests that mean age was approximately 48 years (±13.12), predominantly female patients (80.9%) and married or in a consensual union (42.5%) or single patients (34.4%) [see Supplementary Table S1, as seen in previous publications (12)].

### 3.2. Quality of life (EQ-5D-5L questionnaire), disability (Sheehan Disability Scale – SDS) and work-impairment (WPAI) in TRD patients over a 1-year follow-up

#### 3.2.1. Quality of life – EQ-5D-5L questionnaire

Table 1 presents the results of quality of life assessed through EQ-5D-5L questionnaire, collected at visit 1 for all TRD patients and at the end of study visit (TRD patients included in phase 2 dataset). Approximately 43.5 of the patients reported having problems walking at visit 1 and 33.5% at the end of study. Similarly, 43.7% reported having problems washing or dressing themselves at visit 1 and 27.7% of the phase 2 patients at the end of study. Around 87% at visit 1 and 56.7% at end of study visit of all TRD patients revealed problems doing their usual activities. Approximately 77% of the patients reported having pain or discomfort, while at the end of study the percentages were 57.9%. Almost 3% of the TRD patients did not feel anxious or depressed and 30.9% were severely anxious or depressed at visit 1. At the end of study almost three-quarters (74.4%) reported some anxiety or depression feelings, and the proportion of severe anxious or depressed patients was 12.9%. The patients rated their health at visit 1 with a mean of 50.0 points and 67.1 at the end of study visit, showing statistically significant differences (*p* < 0.0001) among TRD patients. Overall, although some improvement was observed from visit 1 to end of study visit, the proportion of patients with relevant symptoms is still substantial in all domains.

**Table 1:** Quality of life – EQ-5D-5L questionnaire over a 1-year follow-up of TRD patients – values per dimension.

	Visit 1 (*n* = 430)	End of study (*n* = 328)
Mobility, *n* (%)		
I have no problems walking	243 (56.5%)	218 (66.5%)
I have slight problems walking	81 (18.8%)	47 (14.3%)
I have moderate problems walking	80 (18.6%)	47 (14.3%)
I have severe problems walking	25 (5.8%)	16 (4.9%)
I am unable to walk	1 (0.2%)	0
Self-care, *n* (%)		
I have no problems washing/dressing myself	242 (56.3%)	237 (72.3%)
I have slight problems washing/dressing myself	87 (20.2%)	44 (13.4%)
I have moderate problems washing/dressing myself	74 (17.2%)	39 (11.9%)
I have severe problems washing or dressing myself	27 (6.3%)	7 (2.1%)
I am unable to wash or dress myself	0	1 (0.3%)
Usual activities, *n* (%)		
I have no problems doing my usual activities	59 (13.7%)	142 (43.3%)
I have slight problems doing my usual activities	97 (22.6%)	89 (27.1%)
I have moderate problems doing my usual activities	184 (42.8%)	66 (20.1%)
I have severe problems doing my usual activities	73 (17.0%)	27 (8.2%)
I am unable to do my usual activities	17 (4.0%)	4 (1.2%)
Pain/discomfort, *n* (%)		
I have no pain or discomfort	99 (23.0%)	138 (42.1%)
I have slight pain or discomfort	106 (24.7%)	81 (24.7%)
I have moderate pain or discomfort	124 (28.8%)	61 (18.6%)
I have severe pain or discomfort	83 (19.3%)	33 (10.1%)
I have extreme pain or discomfort	18 (4.2%)	15 (4.6%)
Anxiety/depression, *n* (%)		
I am not anxious or depressed	12 (2.8%)	84 (25.6%)
I am slightly anxious or depressed	58 (13.5%)	117 (35.7%)
I am moderately anxious or depressed	175 (40.7%)	61 (18.6%)
I am severely anxious or depressed	133 (30.9%)	42 (12.8%)
I am extremely anxious or depressed	52 (12.1%)	24 (7.3%)
Health in the current day^a^		
Mean (Standard deviation)	50.02 (21.09)	67.12 (24.20)
Minimum-Maximum	0.00–100.00	0.00–100.00
EQ-5D-3L score^b^		
Mean (Standard deviation)	0.62 (0.17)	0.75 (0.21)
Minimum-Maximum	−0.06 (1.00)	0.08 (1.00)
Score recoded as categorical variable, *n* (%)		
Worst health status (score < 0.403)	51 (11.9%)	31 (9.5%)
Higher health status (score ≥ 0.403)	379 (88.1%)	297 (90.5%)
Total	430	328

TRD, Treatment resistant depression. ^a^Longitudinal analysis (Generalized estimating equation GEE model) *B* = 1.371, Confidence Interval 95% = [1.160, 1.582], *p* = <0.0001. ^b^*B* = 0.010, Confidence Interval 95% = [0.008, 0.012], *p* = <0.0001.

The EQ-5D-3L score was computed and the mean value for all TRD patients at visit 1 was 0.62 points and 0.75 at the end of study visit. A statistically significant difference (*p* < 0.0001) was observed among phase 2 patients, with a mean monthly variation of 0.01 points.

#### 3.2.2. Sheehan Disability Scale

Table 2 reports on the results for disability as assessed through the Sheehan Disability Scale. At visit 1, 17.3% of the patients reported that symptoms extremely disrupted their work/school, 17.4% of the patients reported the symptoms extremely disrupted their social life/leisure activities, while 10.9% reported that symptoms extremely disrupted their family life/home responsibilities (Table 2). At the end of the study, this intensity of symptoms was reported by 11.9, 10.3, and 5.1%, respectively. Overall, symptoms persisted in at least 80% of the patients at last visit, indicating a significant disability.

**Table 2:** Sheehan Disability Scale (SDS).

	Visit 1 (*n* = 430)	Visit 3 (*n* = 349)	End of study (*n* = 332)
The symptoms have disrupted your work /school, *n* (%)			
Not at all	13 (3.8%)	22 (9.6%)	42 (17.8%)
Mildly	33 (9.7%)	54 (23.7%)	68 (28.8%)
Moderately	95 (27.9%)	89 (39.0%)	66 (28.0%)
Markedly	141 (41.3%)	47 (20.6%)	32 (13.6%)
Extremely	59 (17.3%)	16 (7.0%)	28 (11.9%)
Total	341	228	236
Mean (Standard deviation)	6.63 (2.70)	4.84 (2.90)	4.25 (3.28)
Minimum-Maximum	0.00–10.00	0.00–10.00	0.00–10.00
The symptoms have disrupted your social life/leisure activities, *n* (%)			
Not at all	9 (2.1%)	30 (8.7%)	56 (16.9%)
Mildly	25 (5.8%)	68 (19.7%)	94 (28.4%)
Moderately	126 (29.3%)	129 (37.3%)	83 (25.1%)
Markedly	195 (45.3%)	87 (25.1%)	64 (19.3%)
Extremely	75 (17.4%)	32 (9.2%)	34 (10.3%)
Total	430	346	331
Mean (Standard deviation)	7.02 (2.35)	5.16 (2.93)	4.44 (3.31)
Minimum-Maximum	0.00 (10.00)	0.00 (10.00)	0.00 (10.00)
The symptoms have disrupted your family life/home responsibilities, *n* (%)			
Not at all	12 (2.8%)	34 (9.9%)	61 (18.4%)
Mildly	42 (9.8%)	86 (24.9%)	98 (29.6%)
Moderately	153 (35.6%)	119 (34.5%)	82 (24.8%)
Markedly	176 (40.9%)	86 (24.9%)	73 (22.1%)
Extremely	47 (10.9%)	20 (5.8%)	17 (5.1%)
Total	430	345	331
Mean (Standard deviation)	6.41 (2.46)	4.89 (2.86)	4.07 (3.12)
Minimum-Maximum	0.00	0.00	0.00
Total score^a^			
*N*	341	228	236
Mean (Standard deviation)	20.30 (6.50)	14.81 (7.97)	12.88 (9.06)
Minimum-Maximum	0.00–30.00	0.00–30.00	0.00–30.00
On how many days in the past 7 days did your symptoms cause you to miss school or work or leave you unable to carry out your normal daily responsibilities			
*N*	430	346	330
Mean (Standard deviation)	2.36 (2.62)	1.20 (1.98)	1.05 (1.90)
Minimum-Maximum	0.00–9.00	0.00–7.00	0.00–7.00
On how many days in the past 7 days did you feel so impaired by your symptoms, that even though you went to school or work or had other daily responsibilities, your productivity was reduced			
*N*	430	346	330
Mean (Standard deviation)	2.45 (2.30)	1.96 (2.17)	1.60 (2.10)
Minimum-Maximum	0.00–7.00	0.00–7.00	0.00–7.00

TRD, Treatment resistant depression. Total values for each scale/dimension vary based on the responses collected at the sites. ^a^Longitudinal analysis (Generalized estimating equation GEE model) *B* = −0.548, Confidence Interval 95% = [−0.640; −0.456], *p* = <0.0001.

The mean total score of SDS was 20.3 in TRD patients at visit 1, while in visit 3 TRD patients presented a mean score of 14.8 points and 12.9 points at end of study visit. Statistically significant differences were identified (*p* < 0.0001) in the longitudinal analysis.

#### 3.2.3. Work productivity and activity impairment questionnaire: depression (WPAI:D)

Table 3 present the results of the WPAI questionnaire to identify work impairment. In the 7 days prior to visit 1, the depression led to a median of 17.7% of work time missed, 60.0% impairment while working, 64.0% of overall work impairment and 70.0% of activity impairment. At the end of the study, the median values among TRD patients included in phase 2 were 0.0, 30.0, 30.0 and 40.0% respectively, a significant impairment in most dimensions after a 1-year of SOC with specialized treatment and physicians.

**Table 3:** Work productivity and activity incapacity questionnaire: depression (WPAI:D).

	Visit 1 (*n* = 430)	Visit 2 (*n* = 368)	Visit 3 (*n* = 349)	Visit 4 (*n* = 335)	End of study (*n* = 332)	Longitudinal analysis GEE model	
Percent work time missed due to depression (%)							
*N*	137	114	117	113	117		
Mean	29.30	17.57	16.49	13.67	13.81		
Median	17.65	4.88	3.57	0.00	0.00	B	−1.228
Standard deviation	34.06	26.35	26.96	23.45	28.07	95% CI	[−1.782; −0.673]
Minimum	0.00	0.00	0.00	0.00	0.00	Value of *p*	<0.0001
Maximum	100.00	100.00	100.00	100.00	100.00		
Percent impairment while working due to depression (%)							
*N*	122	109	110	111	111		
Mean	51.89	38.53	37.36	33.33	30.90		
Median	60.00	40.00	40.00	30.00	30.00	B	−1.616
Standard deviation	26.61	26.52	29.36	26.33	27.88	95% CI	[−2.151; −1.082]
Minimum	0.00	0.00	0.00	0.00	0.00	Value of *p*	<0.0001
Maximum	100.00	100.00	100.00	90.00	100.00		
Percent overall work impairment due to depression (%)							
*N*	121	109	110	111	111		
Mean	59.49	45.22	42.02	38.87	33.98		
Median	64.00	47.74	40.00	40.00	30.00	B	−2.021
Standard deviation	27.76	28.88	31.27	29.70	30.09	95% CI	[−2.593; −1.448]
Minimum	0.00	0.00	0.00	0.00	0.00	Value of *p*	<0.0001
Maximum	100.00	100.00	100.00	99.85	100.00		
Percent activity impairment due to depression (%)							
*N*	429	365	346	335	331		
Mean	66.36	52.36	49.05	44.45	41.12		
Median	70.00	50.00	50.00	40.00	40.00	B	−1.977
Standard deviation	24.29	27.80	28.96	30.15	31.55	95% CI	[−2.268; −1.686]
Minimum	0.00	0.00	0.00	0.00	0.00	Value of *p*	<0.0001
Maximum	100.00	100.00	100.00	100.00	100.00		

TRD, Treatment resistant depression; GEE, Generalized estimating equation; 95%CI, 95% Confidence interval.

Results based on phase 2 patients, showed that all items of productivity and impairment described above presented a statistically significant result on the longitudinal analysis, with a mean monthly variation of-1.2, −1.6%, −2.0%, and −2.0%, respectively. Regardless, the percentage of work impairment at end of study visit remains high and significant for a relevant proportion of patients-in some cases over 40% of the sample (Table 3).

### 3.3. Depression severity in TRD patients over a 1-year follow-up

#### 3.3.1. Patient Health Questionnaire

PHQ-9 was used to assess depression severity and impact on instrumental activities of daily living. At the end of study visit, 31% of the sample still characterized the impact of depression as very difficult/extremely difficult after 1-year under SOC. The mean total score of PHQ-9 at visit 1 was 17.4 points and, according to this scale, 28.9% had moderately severe depression and 40.1% severe depression. The mean score at the end of study for phase 2 patients was 10.8 points but 18.8% of these patients were still classified as severe, and 16.4% as moderately severe. Statistically significant differences (*p* < 0.0001) across the visits were identified, with a mean monthly variation of-0.5 points (Table 4).

**Table 4:** Reported analysis of TRD patients 1-year follow-up with questionnaire on patient’s health (PHQ-9).

	Visit 1 (*n* = 430)	Visit 3 (*n* = 349)	End of study (*n* = 332)	Longitudinal analysis GEE model	
Total score^a^					
*N*	429	348	329		
Mean	17.44	12.68	10.77		
Median	18.00	13.00	9.00	B	−0.543
Standard deviation	5.60	7.16	8.00	95% CI	[−0.612; −0.474]
Minimum	2.00	0.00	0.00	Value of *p*	<0.0001
Maximum	27.00	27.00	27.00		
Depression severity, *n* (%)					
None (0–4)	5 (1.2%)	56 (16.1%)	96 (29.2%)		
Mild (5–9)	33 (7.7%)	75 (21.6%)	69 (21.0%)		
Moderate (10–14)	95 (22.1%)	69 (19.8%)	48 (14.6%)		
Moderately severe (15–19)	124 (28.9%)	77 (22.1%)	54 (16.4%)		
Severe (20–27)	172 (40.1%)	71 (20.4%)	62 (18.8%)		
Total	429	348	329		
If you checked off any problems, how difficult have these problems made it for you to do your work, take care of things at home, or get along with other people?, *n* (%)					
Not difficult at all	7 (1.6%)	38 (11.0%)	72 (21.9%)		
Somewhat difficult	135 (31.5%)	168 (48.4%)	155 (47.1%)		
Very difficult	226 (52.7%)	115 (33.1%)	76 (23.1%)		
Extremely difficult	61 (14.2%)	26 (7.5%)	26 (7.9%)		
Total	429	347	329		

^a^Total score range between 0 and 27 and higher values indicate higher depression severity. MDD, Major Depressive Disorder; TRD, treatment resistant depression; GEE, Generalized estimating equation; 95%CI, 95% Confidence interval. Total values for each scale/dimension vary based on the responses collected at the sites.

#### 3.3.2. PHQ-9 versus MADRS scores

As presented in Table 5, there was a statistically significant and positive correlation between the MADRS and PHQ-9 scores at visit 1 and also at the end of study visit, with positive and strong correlation at visit 1 (rs = 0.639, *p* < 0.0001) and again at end of study visit (rs = 0.894, *p* < 0.0001).

**Table 5:** Correlation between PHQ-9 and MADRS scores at visit 1 and visit 5.

	Visit 1 (*n* = 430)	End of study (*n* = 332)
PHQ-9 *vs* MADRS		
*N*	429	329
Spearman correlation coefficient	0.639	0.894
Value of *p*	<0.001	<0.001

MDD, Major Depressive Disorder; TRD, Treatment resistant depression.

These results are consistent with the ones found and previously published (12) for the main clinical outcomes of the TRAL project, in which severity of depression was still present in a significant proportion of TRD patients. Moreover, the proportion of patients achieving response was well under 50%.

### 3.4. Results overview

At the end of study visit, the impact of TRD was still considered to be significant in the patients’ assessment in multiple dimensions. Work impairment is a clear outcome in this context. The statistically significant reduction of disability scores should be noted, but the proportion of patients still reporting noticeable disability after one year of follow-up should not be underestimated. Lastly, the impact of TRD in quality of life remains significant at the end of study, although the study found a statistically significant reduction in the score over the longitudinal analysis.

## 4. Discussion

TRAL was a comprehensive project that provided relevant and much needed data on the epidemiological characterization of TRD in the region. Beyond this, clinical features and outcomes in this setting were also the focus of the cross-sectional (39, 48) and longitudinal analyses (12). Although PROs have increased in importance for RWD generation, these were commonly not included and longitudinally analyzed. The most salient results of the current longitudinal analysis show that regardless of the statistically significant reduction in some of the dimensions, the burden of the disease after 1-year of SOC remains significant, namely in severity of the symptoms, disability, quality of life and work-impairment. Given this, and in accordance with the cross-sectional stage of this project, the unmet need concerning the lack of evidence in LatAm on epidemiological data and burden of the disease in TRD patients was addressed.

The first key finding from the study identifies another unmet need in the region. Current SOC does not accomplish the level of psychosocial and clinical outcomes desired in TRD patients, even under direct supervision of specialized psychiatrists and on permanent clinical follow-up at reference sites. This had already been stated in other TRAL publications (12), but the present results obtained for QoL, disability and work impairment underline these conclusions. SOC fails to provide TRD patients with the necessary return to pre-disease status (34, 35). Previous research identified diminished proportion of TRD patients that achieved a clinical response (slightly over 40%). Present results corroborate such claims since almost 50% of patients under SOC after 1-year follow-up still present moderate to severe depressive symptomatology (PHQ-9). Other publications identified that the severity clinically depressive symptoms was associated with a higher burden of the disease (49). Concomitantly, TRD patients incur much higher healthcare expenditure compared to MDD and to controls. A European study found that TRD patients elicit significantly higher healthcare costs, with worse quality of life and higher work productivity costs (34). Results obtained with SOC were subpar and hence newer therapies with better efficacy are needed.

The simultaneous utilization of PHQ-9 and MADRS showed a high correlation. Research with novel therapies for the treatment of TRD had shown the consistency of positive clinical outcomes using both scales as efficacy tools (50, 51). Concerning the lack of consensus on both the clinical definition of TRD (8, 52) and clinical outcomes (response, relapse and remission) assessment, consistent results between two instruments increases the methodological soundness.

The present results obtained in QoL, disability and Work-impairment instruments are also of note. Although there was a statistically significant improvement in QoL (EQ-5D) over time, the scores obtained are still distanced from the normative scores (47, 53). TRD patients’ results after 1-year of SOC are closer to MDD patients reference values, far from the desired outcome, representing a very negative outcome in QoL. The same can be stated for disability. On average, these patients remain with moderate disability after 1-year. However, the improvement seems to be statistically significant. Concerning work-impairment, improvement is visible in a portion of the sample, but the detrimental impact in the work performance remains, in line with previous research (54, 55). This study reveals that despite improvements observed in quality of life, work impairment and disability outcomes after 1-year of SOC among TRD patients, the management of patients still needs to be optimized.

The primary TRAL publication focused on the clinical outcomes of SOC treatment, which provided a solid depiction of current trends and unmet need in LatAm, this study has several strengths. Beyond the use of clinically validated instruments, sampling was adequately defined, and the diversity of reference centers and locations attest for the generalization potential to LatAm, although the same is not recommended at country-level (12). The use of real-world data is both a positive feature and a challenge. The variability of treatment protocols and therapies in the study centers limit the inferences that can be performed on the effectiveness of a specific treatment. The same can be said for the clinical presentation of the patients, as disease severity at baseline or the time from diagnosis were not considered as co-variates in the longitudinal analysis. Nevertheless, this also increases the heterogeneity of patient profiles and allows for a broader characterization of real-world practice. The drop-out from baseline to the end-of-study visit is around 23%, which although expected in these patients may have artificially incremented the efficacy of SOC.

Future TRAL publications will increase the knowledge in this regard, but currently available publications and the present results clearly underline the unmet needs concerning current SOC. Previous publications (39) showed the increased burden in all analyzed dimensions-from an economic to a more humanistic perspective-in the lives of TRD compared to other MDD (56). Healthcare decision-makers should analyze these data and promote a set of measures to increase the availability of other treatment options, since the burden of TRD is not only clear in a clinical standpoint, but also poses a significant threat for quality of life and overall living standards of the patients afflicted by this condition. Irrespective of the severity of the clinical presentation of TRD, this prevalent condition with a refractory nature should always be considered an often life-threatening and serious condition.

The current research protocol, as previously stated, was developed to address both epidemiological and burden of disease characterization in TRD patients in LatAm. The current results add to the previously published (12, 48, 57, 58) by addressing different dimensions and for the longitudinal analysis of the outcomes. There are some limitations inherent to the study design that should be mentioned. The sample is not necessarily representative of the region nor of each of the individual countries analyzed since only patients followed regularly and attending routine medical appointments were included. Moreover, and given the discussion surrounding the definition of TRD, and while considering that the definition used in this manuscript was aligned with reference literature (8, 11, 59), it should be assumed that this condition is underdiagnosed even in MDD patients followed at reference treatment centers. The heterogeneity of patient’s profile and clinical management provided by this observational design and the study population is both a strong feature and a limitation of this study. On one hand, it clearly depicts the real-world context of TRD, but on the other hand may have produced data that is incomplete or biased, given the differences between countries, regions and centers. Future research should consider aspects such as a representative sample of the region-which can be achieved by including other countries and a larger sample-, to analyze differences in the outcomes between treatment regimens, and to compare TRD and non-TRD patients over time, which can provide essential evidence of the added burden of TRD and on the clinical complexity of managing patients clinically diagnosed with this condition.

Concerning the implications for the clinical setting, diagnosis remains a concern and this should be addressed. To achieve better diagnostic outcomes, further efforts should be placed in a practical and applicable operational definition of TRD. The considerable burden of TRD, the heterogeneity of patient’s profile, and the diversity of therapeutic options should also lead to a revised approach to patient management, in which individualized, and patient-centric strategies must be considered. Given the outcomes identified in both clinical and non-clinical dimensions in the TRAL project, and lack of response, treatment switching and optimization seems to be a necessity.

Some important measures to decrease the burden of TRD include patient education, increased treatment adherence and seeking earlier help after symptom onset. This has the potential, alongside the training of primary care physicians and more synergies between primary care and psychiatry, to increase early diagnosis and shorten the time from symptom onset to treatment initiation. Given the overall impact of MDD, the significant proportion of patients developing TRD, and both the humanistic and economic burden of this condition, research on this subject should be prioritized to identify best practices and address the major identifies unmet needs.

## 5. Conclusion

Overall, the TRAL study highlighted the burden of TRD in LatAm on multiple dimensions, most notably including severe impact in TRD patients’ psychological adjustment, work performance and overall independence in conducting instrumental activities of daily living, as well as low general QoL. After a 1 year of follow-up in TRD patients under SOC, the burden of the disease is still significant, and depressive symptoms afflict a significant proportion of patients. Moreover, the improvement of clinical symptoms obtained with SOC is insufficient. The use of PROs is essential to provide a good depiction of the burden of the disease, and to highlight the increased challenges posed by TRD. The impact of the unmet needs in the treatment of TRD may be reduced with the introduction of novel therapies, as well as earlier diagnoses. Present results should constitute a catalyst for a more profound and robust intervention by all relevant stakeholders involved in the mental healthcare ecosystem. Policy makers, as well as all involved in the process must cooperate to find ways to provide patients with better therapies, facilitating the return to their original condition prior to TRD.

## Data availability statement

The raw data supporting the conclusions of this article will be made available by the authors, without undue reservation.

## Ethics statement

The studies involving human participants were reviewed and approved by Argentina: 1. Comité Independiente de Ética de Investigación en Salud Prof. Dr. Marcelino Rusculleda, 2. Comité de Bioética e Investigación de la Fundación para el Estudio y Tratamiento de las Enfermedades Mentales (FETEM), 3. CAICI – CIAP Instituto Centralizado de Asistencia e Investigación Clínica Integral – Centro de Investigación y Asistencia en Psiquiatría, 4. Instituto Médico Platense S.A., 5. Comité Independiente de Ética para Ensayo en Farmacología Clínica. Fundación de Estudios Farmacológicos y de Medicamentos Prof. Luis M. Zeiher. Brazil: 1. Comissão Nacional de Ética em Pesquisa – CONEP, 2. Comitê de Ética em Pesquisa Investiga – Instituto de Pesquisa, 3. Comitê de Ética em Pesquisa Instituto de Psiquiatria da Universidade Federal do Rio de Janeiro – IPUB/UFRJ, 4. Comitê de Ética em Pesquisa Secretaria de Estado da Saúde de Santa Catarina – SES, 5. Comitê de Ética em Pesquisa Hospital Universitário Walter Cantídio da Universidade Federal do Ceará – UFC, 6. Comitê de Ética em Pesquisa Instituto de Neurologia de Curitiba, 7. Comitê de Ética em Pesquisa Universidade Federal de Minas Gerais – UFMG, 8. Comitê de Ética em Pesquisa Faculdade de Medicina da Universidade Federal de Pelotas – UPEL, 9. Comitê de Ética em Pesquisa Hospital Universitário Professor Edgard Santos-UFBA, 10. Comitê de Ética em Pesquisa Hospital de Clínicas de Porto Alegre da Universidade Federal do Rio Grande do Sul – UFRGS, 11. Comitê de Ética em Pesquisa Hospital das Clínicas da Faculdade de Medicina da Universidade de São Paulo – USP, 12. Comitê de Ética em Pesquisa Hospital São José. Colombia: 1. C.E.I. Campo Abierto LTDA, 2. E.S.E. Hospital Mental de Antioquia, 3. Clínica CEIC de la Fundación Centro de Investigación Clínica, CIC, 4. C.E.I. Campo Abierto LTDA. Mexico: 1. Investigación Biomédica para el Desarrollo de Fármacos, S.A. de C.V, 2. Instituto Nacional de Neurología y Neurocirugía Manuel Velazco Suárez, 3. Hospital La Misión, S.A. de C.V., 4. Instituto Nacional de Psiquiatría Ramón de la Fuente Muñiz, 5. Hospital Central Dr. Ignacio Morones Prieto. The patients/participants provided their written informed consent to participate in this study. The studies were conducted in accordance with the local legislation and institutional requirements.

## Author contributions

KR, GB, CL, AN, AM, CB-P, SP, and AP contributed to the conception, design, analysis, interpretation of available data, wrote the manuscript, and reviewed drafts. All authors contributed to the article and approved the submitted version.

## References

[1] Malhi GS Mann JJ. Depression. Lancet. (2018) 392:2299–312. doi: 10.1016/S0140-6736(18)31948-2 30396512

[2] World Health Organization. Depression and other common mental disorders: Global health estimates. (2017). 1–24. World Health Organization: Geneva.

[3] Kennedy SH. Core symptoms of major depressive disorder: relevance to diagnosis and treatment. Dialogues Clin Neurosci. (2008) 10:271–7. doi: 10.31887/DCNS.2008.10.3/shkennedy, PMID: 18979940 PMC3181882

[4] Mrazek DA Hornberger JC Altar CA Degtiar I. A review of the clinical, economic, and societal burden of treatment-resistant depression: 1996–2013. Psychiatr Serv. (2014) 65:977–87. doi: 10.1176/appi.ps.201300059, PMID: 24789696

[5] Gaynes BN Warden D Trivedi MH Wisniewski SR Fava M Rush AJ. What did STAR*D teach us? Results from a large-scale, practical, clinical trial for patients with depression. Psychiatr Serv. (2009) 60:1439–45. doi: 10.1176/ps.2009.60.11.1439, PMID: 19880458

[6] John Rush A Trivedi MH Wisniewski SR Nierenberg AA Stewart JW Warden D. Acute and longer-term outcomes in depressed outpatients requiring one or several treatment steps: a STAR*D report. Am J Psychiatry. (2006) 163:1905–17. doi: 10.1176/ajp.2006.163.11.1905 17074942

[7] Stegenga BT Kamphuis MH King M Nazareth I Geerlings MI. The natural course and outcome of major depressive disorder in primary care: the PREDICT-NL study. Soc Psychiatry Psychiatr Epidemiol. (2012) 47:87–95. doi: 10.1007/s00127-010-0317-9, PMID: 21057769 PMC3249585

[8] Conway CR George MS Sackeim HA. Toward an evidence-based, operational definition of treatment-resistant depression: when enough is enough. JAMA Psychiatry. (2017) 74:9–10. doi: 10.1001/jamapsychiatry.2016.2586, PMID: 27784055

[9] Bennabi D Charpeaud T Yrondi A Genty JB Destouches S Lancrenon S. Clinical guidelines for the management of treatment-resistant depression: French recommendations from experts, the French Association for Biological Psychiatry and Neuropsychopharmacology and the fondation FondaMental. BMC Psychiatry. (2019) 19:262–12. doi: 10.1186/s12888-019-2237-x, PMID: 31455302 PMC6712810

[10] Li CT. Overview of treatment-resistant depression. Prog Brain Res. (2023) 278:7. doi: 10.1016/BS.PBR.2023.03.007 37414489

[11] Sforzini L Worrell C Kose M Anderson IM Aouizerate B Arolt V. A Delphi-method-based consensus guideline for definition of treatment-resistant depression for clinical trials. Mol Psychiatry. (2022) 27:1286–99. doi: 10.1038/s41380-021-01381-x, PMID: 34907394 PMC9095475

[12] Caldieraro MA Tung TC Agudelo Baena LM Vilapriño Duprat M Corral RM Alviso de la Serna LD. Depression and suicidality severity among TRD patients after 1-year under standard of care: findings from the TRAL study, a multicenter, multinational, observational study in Latin America. Rev Psiquiatr Salud Ment. (2022). doi: 10.1016/j.rpsm.2022.06.002 38591721

[13] FDA. FDA briefing document psychopharmacologic drugs advisory committee (PDAC) and drug safety and risk management (DSaRM) Advisory Committee Meeting, November 2, 2018 (2019). 1–135.

[14] ICER. *Esketamine for the treatment of treatment-resistant depression: Effectiveness and value*. Final evidence report prepared for CEPAC. (2019).

[15] Wilkinson ST Howard DH Busch SH. Psychiatric practice patterns and barriers to the adoption of Esketamine. JAMA. (2019) 322:1039–40. doi: 10.1001/jama.2019.10728, PMID: 31373610

[16] Perugi G Calò P De Filippis S Rosso G Vita A Adami M. Clinical features and outcomes of 124 Italian patients with treatment resistant depression: a real-world, prospective study. Front Psychiatry. (2021) 12:769693. doi: 10.3389/fpsyt.2021.769693, PMID: 34803777 PMC8603563

[17] Heerlein K Perugi G Otte C Frodl T Degraeve G Hagedoorn W. Real-world evidence from a European cohort study of patients with treatment resistant depression: treatment patterns and clinical outcomes. J Affect Disord. (2021) 290:334–44. doi: 10.1016/j.jad.2021.03.073, PMID: 34044256

[18] Park HJ Park CM Woo JM Shin JY Lee EK Kwon SH. Real-world data analysis of the clinical and economic burden and risk factors in patients with major depressive disorder with an inadequate response to initial antidepressants. J Med Econ. (2021) 24:589–97. doi: 10.1080/13696998.2021.1918922, PMID: 33879031

[19] De DZ Robins PL Dannhauer M Haugen LM Port JD Croarkin PE. Optimizing TMS coil placement approaches for targeting the dorsolateral prefrontal cortex in depressed adolescents: an electric field modeling study. Biomedicine. (2023) 11:2320. doi: 10.3390/biomedicines11082320, PMID: 37626817 PMC10452519

[20] Bellini H Cretaz E Carneiro AM da Silva PHR dos Santos LA Gallucci-Neto J. Magnetic waves vs. electric shocks: a non-inferiority study of magnetic seizure therapy and electroconvulsive therapy in treatment-resistant depression. Biomedicine. (2023) 11:2150. doi: 10.3390/biomedicines11082150 PMC10452083 37626647

[21] Vasiliu O. Efficacy, Tolerability, and safety of Toludesvenlafaxine for the treatment of major depressive disorder—a narrative review. Pharmaceuticals. (2023) 16:411. doi: 10.3390/ph16030411, PMID: 36986510 PMC10051807

[22] Dean RL Hurducas C Hawton K Spyridi S Cowen PJ Hollingsworth S. Ketamine and other glutamate receptor modulators for depression in adults with unipolar major depressive disorder. Cochrane Database Syst Rev. (2021) 2021:CD011612. doi: 10.1002/14651858.CD011612.pub3, PMID: 34510411 PMC8434915

[23] Wang Y-T Wang X-L Feng S-T Chen N-H Wang Z-Z Zhang Y. Novel rapid-acting glutamatergic modulators: targeting the synaptic plasticity in depression. Pharmacol Res. (2021) 171:105761. doi: 10.1016/j.phrs.2021.105761, PMID: 34242798

[24] Khoodoruth MAS Estudillo-Guerra MA Pacheco-Barrios K Nyundo A Chapa-Koloffon G Ouanes S. Glutamatergic system in depression and its role in Neuromodulatory techniques optimization. Front. Psychiatry. (2022) 13:6918. doi: 10.3389/fpsyt.2022.886918, PMID: 35492692 PMC9047946

[25] Henter ID De Sousa RT Zarate CA. Glutamatergic modulators in depression. Harv Rev Psychiatry. (2018) 26:307–19. doi: 10.1097/HRP.0000000000000183, PMID: 29465478 PMC6102095

[26] Vila-Merkle H González-Martínez A Campos-Jiménez R Martínez-Ricós J Teruel-Martí V Lloret A. Sex differences in amygdalohippocampal oscillations and neuronal activation in a rodent anxiety model and in response to infralimbic deep brain stimulation. Front Behav Neurosci. (2023) 17:2163. doi: 10.3389/fnbeh.2023.1122163, PMID: 36910127 PMC9995972

[27] Tanaka M Telegdy G. Involvement of adrenergic and serotonergic receptors in antidepressant-like effect of urocortin 3 in a modified forced swimming test in mice. Brain Res Bull. (2008) 77:301–5. doi: 10.1016/j.brainresbull.2008.08.012, PMID: 18793704

[28] Adamu MJ Qiang L Nyatega CO Younis A Kawuwa HB Jabire AH. Unraveling the pathophysiology of schizophrenia: insights from structural magnetic resonance imaging studies. Front Psychiatry. (2023) 14:1188603. doi: 10.3389/fpsyt.2023.1188603 37275974 PMC10236951

[29] Allen CM Bray C. Improving patient-centered care for veterans with treatment-resistant depression using shared decision-making tools. J Am Psychiatr Nurses Assoc. (2023) 29:7–14. doi: 10.1177/10783903221141885 36510357

[30] Shillington AC Langenecker SA Shelton RC Foxworth P Allen L Rhodes M. Development of a patient decision aid for treatment resistant depression. J Affect Disord. (2020) 275:299–306. doi: 10.1016/j.jad.2020.07.014, PMID: 32734922

[31] Johnston KM Powell LC Anderson IM Szabo S Cline S. The burden of treatment-resistant depression: a systematic review of the economic and quality of life literature. J Affect Disord. (2019) 242:195–210. doi: 10.1016/j.jad.2018.06.045, PMID: 30195173

[32] Pilon D Sheehan JJ Szukis H Morrison L Zhdanava M Lefebvre P. Is clinician impression of depression symptom severity associated with incremental economic burden in privately insured US patients with treatment resistant depression? J Affect Disord. (2019) 255:50–9. doi: 10.1016/j.jad.2019.04.100, PMID: 31128505

[33] Lundberg J Cars T Lööv SÅ Söderling J Sundström J Tiihonen J. Association of Treatment-Resistant Depression with Patient Outcomes and Health Care Resource Utilization in a population-wide study. JAMA Psychiatry. (2023) 80:167–75. doi: 10.1001/JAMAPSYCHIATRY.2022.3860, PMID: 36515938 PMC9856735

[34] Jaffe DH Rive B Denee TR. The humanistic and economic burden of treatment-resistant depression in Europe: a cross-sectional study. BMC Psychiatry. (2019) 19:247–11. doi: 10.1186/s12888-019-2222-4, PMID: 31391065 PMC6686569

[35] Dibernardo A Lin X Zhang Q Xiang J Lu L Jamieson C. Humanistic outcomes in treatment resistant depression: a secondary analysis of the STAR∗D study. BMC Psychiatry. (2018) 18:1–8. doi: 10.1186/s12888-018-1920-7, PMID: 30373547 PMC6206859

[36] Amos TB Tandon N Lefebvre P Pilon D Kamstra RL Pivneva I. Direct and indirect cost burden and change of employment status in treatment-resistant depression: a matched-cohort study using a us commercial claims database. J Clin Psychiatry. (2018) 79:11725. doi: 10.4088/JCP.17m11725, PMID: 29474009

[37] Gillain B Degraeve G Dreesen T De Bruecker G Buntinx E Beke D. Real-world treatment patterns, outcomes, resource utilization and costs in treatment-resistant major depressive disorder: PATTERN, a retrospective cohort study in Belgium. Pharmacoecon Open. (2022) 6:293–302. doi: 10.1007/s41669-021-00306-2, PMID: 34782984 PMC8864045

[38] Chow W Doane MJ Sheehan J Larry A Le H. Economic burden among patients with major depressive disorder: an analysis of healthcare resource use, work productivity, and direct and indirect costs by depression severity. Am J Manag Care. (2019) 76:155–62. doi: 10.4088/JCP.14m09298

[39] Soares B Kanevsky G Teng CT Pérez-Esparza R Bonetto GG Lacerda ALT. Prevalence and impact of treatment-resistant depression in Latin America: a prospective, Observational Study. Psychiatric Quarterly. (2021) 92:1797–815. doi: 10.1007/s11126-021-09930-x, PMID: 34463905 PMC8531108

[40] Pérez-Sola V Roca M Alonso J Gabilondo A Hernando T Sicras-Mainar A. Economic impact of treatment-resistant depression: a retrospective observational study. J Affect Disord. (2021) 295:578–86. doi: 10.1016/j.jad.2021.08.036, PMID: 34509073

[41] Montgomery SA Asberg M. A new depression scale designed to be sensitive to change. Br J Psychiatry. (1979) 134:382–9. doi: 10.1192/bjp.134.4.382 444788

[42] Kroenke K Spitzer RL Williams JBW. The PHQ-9: validity of a brief depression severity measure. J Gen Intern Med. (2001) 16:606–13. doi: 10.1046/j.1525-1497.2001.016009606.x, PMID: 11556941 PMC1495268

[43] Spitzer RL Kroenke K Williams JBW. Validation and utility of a self-report version of PRIME-MD: the PHQ primary care study. J Am Med Assoc. (1999) 282:1737–44. doi: 10.1001/jama.282.18.1737, PMID: 10568646

[44] Reilly MC Zbrozek AS Dukes EM. The validity and reproducibility of a work productivity and activity impairment instrument. PharmacoEconomics. (1993) 4:353–65. doi: 10.2165/00019053-199304050-00006, PMID: 10146874

[45] Sheehan KH Sheehan DV. Assessing treatment effects in clinical trials with the Discan metric of the Sheehan Disability Scale. Int Clin Psychopharmacol. (2008) 23:70–83. doi: 10.1097/YIC.0b013e3282f2b4d6, PMID: 18301121

[46] Herdman M Gudex C Lloyd A Janssen M Kind P Parkin D. Development and preliminary testing of the new five-level version of EQ-5D (EQ-5D-5L). Qual Life Res. (2011) 20:1727–36. doi: 10.1007/s11136-011-9903-x, PMID: 21479777 PMC3220807

[47] van Hout B Janssen MF Feng Y-S Kohlmann T Busschbach J Golicki D. Interim scoring for the EQ-5D-5L: mapping the EQ-5D-5L to EQ-5D-3L value sets. Value Health. (2012) 15:708–15. doi: 10.1016/j.jval.2012.02.008, PMID: 22867780

[48] Corral R Alessandria H Agudelo Baena LM Ferro E Duque X Quarantini L. Suicidality and quality of life in treatment-resistant depression patients in Latin America: secondary interim analysis of the TRAL study. Front Psychiatry. (2022) 13:812938. doi: 10.3389/fpsyt.2022.812938, PMID: 35308889 PMC8924115

[49] Lynch F Dickerson J O’Keeffe-Rosetti M Sengupta S Chow W Jaqueline P. Incremental healthcare costs for persons with treatment resistant depression in managed care organizations. Am J Manag Care. (2020) 1–3.

[50] Bahr R Lopez A Rey JA. Intranasal Esketamine (SpravatoTM) for use in treatment-resistant depression in conjunction with an oral antidepressant. P T. (2019) 44:340–75. PMID: 31160868 PMC6534172

[51] Hudgens S Floden L Blackowicz M Jamieson C Popova V Fedgchin M. Meaningful change in depression symptoms assessed with the Patient Health Questionnaire (PHQ-9) and Montgomery-Åsberg depression rating scale (MADRS) among patients with treatment resistant depression in two, randomized, double-blind, active-controlled trials of Esketamine nasal spray combined with a new Oral antidepressant. J Affect Disord. (2021) 281:767–75. doi: 10.1016/j.jad.2020.11.066, PMID: 33261932

[52] Demyttenaere K Van Duppen Z. The impact of (the concept of) treatment-resistant depression: an opinion review. Int J Neuropsychopharmacol. (2019) 22:85–92. doi: 10.1093/ijnp/pyy052, PMID: 29961822 PMC6368367

[53] Janssen MF Szende A Cabases J Ramos-Goñi JM Vilagut G König HH. Population norms for the EQ-5D-3L: a cross-country analysis of population surveys for 20 countries. Europ J Health Econ. (2019) 20:205–16. doi: 10.1007/s10198-018-0955-5, PMID: 29445941 PMC6438939

[54] Rizvi SJ Grima E Tan M Rotzinger S Lin P McIntyre RS. Treatment-resistant depression in primary care across Canada. Can J Psychiatr. (2014) 59:349–57. doi: 10.1177/070674371405900702, PMID: 25007419 PMC4086317

[55] Congio AC Norcia M Urbano MR Verri WA Vargas Nunes SO. Association of clinical features and biomarkers with treatment-resistant depression. Neurol Psychiatry Brain Res. (2020) 36:32–8. doi: 10.1016/j.npbr.2020.02.004

[56] Kubitz N Mehra M Potluri RC Garg N Cossrow N. Characterization of treatment resistant depression episodes in a cohort of patients from a US commercial claims database. PLoS One. (2013) 8:e76882–9. doi: 10.1371/journal.pone.0076882, PMID: 24204694 PMC3799999

[57] Vázquez Hernández JL Alviso de la Serna LD Cruz C Becerra Palars C Ibarreche Beltran J Kanevsky G. (2023). Prevalence of treatment resistant depression: TRAL study sub analysis in a Mexican sample |. Archivos de Neurociencias. Available at: https://archivosdeneurociencias.org/index.php/ADN/article/view/448 (Accessed June 19, 2023)

[58] Cordoba-Rojas R Ferro E Agudelo Baena LM Kanevsky G Cabrera P. Epidemiology and burden of treatment-resistant depression in Colombia: analysis of the TRAL study. Rev Colomb Psiquiatr. (2023). doi: 10.1016/J.RCP.2023.04.009 41213741

[59] Gaynes BN Asher G Gartlehner G Hoffman V Green J Boland JLL. Definition of treatment-resistant depression in the Medicare population. Technology assessment program – technology assessment; project ID: PSYT0816. Rockville, MD: Agency for Healthcare Research and Quality (US) (2018). 115 p. 30260611

